# Essential in-vitro laboratory diagnostic services provision in accordance with the WHO standards in Guragae zone primary health care unit level, South Ethiopia

**DOI:** 10.1186/s40794-020-0104-x

**Published:** 2020-03-06

**Authors:** Teha Shumbej, Sofia Menu, Teklemichael Gebru, Tadele Girum, Fitsum Bekele, Absra Solomon, Dereje Mesfin, Abdulewhab Jemal

**Affiliations:** 1 Department of Medical Laboratory Sciences, College of Medicine and Health Sciences, Wolkite University, Wolkite, Ethiopia; 2 Department of Medicine, College of Medicine and Health Sciences, Wolkite University, Wolkite, Ethiopia; 3 Department of Public Health, College of Medicine and Health Sciences, Wolkite University, Wolkite, Ethiopia

**Keywords:** Essential in-vitro diagnostics services, PHCU, Guragae zone, Ethiopia

## Abstract

**Introduction:**

Laboratory services are crucial parts of the health system having a great contribution to disease prevention and management. The importance of accurate and reliable laboratory test results is less recognized in developing countries like Ethiopia where most medical decisions are based on clinical judgment. It is time for countries like Ethiopia to not only increase health care coverage but also improve access to essential diagnostic tests. Hence, this proposed study aims to assess essential in-vitro laboratory service provision in accordance with the WHO standards in Guragae Zone primary health care unit level, South Ethiopia.

**Methods:**

Health institution-based cross-sectional study was carried out. 30% randomly selected primary health care units were recruited. Each facility was visited with a WHO checklist by a trained data collector to assess the availability of essential diagnostics service provision. The proportion of available in-vitro diagnostics services was calculated. Results were presented as percentages in tables and figures.

**Result:**

Twenty-one primary health care facilities located in Guragae Zone were assessed between May and July 2019. All surveyed facilities had major gaps in essential test availability. Among essential diagnostic tests listed with WHO like C-reactive protein, lipid profile, Amylase and Lipase, TroponinT/I, hepatitis B e-antigen, IgM-specific antibodies to hepatitis B core antigen, Glucose-6-phosphate dehydrogenase activity, and anti-HIV/p24 rapid test were not provided in any facilities. However, essential diagnostic services like urine dipstick testing, random blood sugar, smear microscopy, and few serological tests were provided at all primary health care units. All surveyed facilities had limited major laboratory equipment and consumables.

**Conclusion and recommendation:**

The present study shows limited access to essential laboratory tests at the primary health care level. Hence, the responsible body should invest to make essential tests accessible at the primary care unit level within the framework of universal health coverage in the study area. The fact that access to essential diagnostic tests is the first key step in improving quality of care; such study has its own efforts to enable the implementation of essential diagnostic lists, and improve access to diagnostics in the country.

## Background

Although both communicable and non-communicable diseases (NCDs) continue to cause millions of deaths and a huge burden of disability every year, access to laboratory services has been a challenge in developing countries [1]. However, when laboratory tests are used optimally, it generates knowledge that facilitates appropriate disease case management and leads to more cost-effective healthcare [2]. Appropriate disease case management at primary health care unit (PHCU) level has paramount importance in the prevention and control of medically important diseases [3].

Increasing access and utilization of adequate laboratory services for diseases could have a major impact on tackling disease burden [4]. World Health Organization (WHO) also advocates for laboratory tests to support appropriate disease management [5]. However, diagnostic laboratories are ill-integrated into the diagnostic and care delivery process in Sub Saharan Africa (SSA) and some common infections can be managed clinically without the need for in-vitro diagnostic tests [6]. Policymakers, clinicians, and the public frequently fail to understand that laboratory diagnosis service is essential to the prevention and treatment of disease [7].

Allocation of resources to diagnostic laboratory services have not been a priority for SSA and access to reliable laboratory diagnostic services are severely limited in these regions, but unreliable and inaccurate laboratory diagnostic tests lead to unnecessary expenditures in a region already plagued by resource shortages and promotes the perception that laboratory services are unhelpful and compromises patient care and leads to misdiagnosis [8]. It has been common practice that diagnosis of diseases mainly based on clinical signs and symptoms in health facilities across Africa [9, 10]. This clinical grounds alone based approach has led to substantial over or under diagnosis [11–13] and unnecessary use of drugs which may lead to pathogen resistance [14].

Laboratory confirmation based disease management may prevent unnecessary treatments, reduce the potential development of drug resistance [15], and encourage health care providers to search for alternative causes for confirmed negative patients [16]. In-vitro diagnostic laboratory services are a component of national disease control and prevention program in most African countries including Ethiopia [17]. Though, diagnostic laboratory services are a critical component of national health systems but face major operational challenges [18]. Lack of access to good-quality diagnostic tests in this region has been a major contributor to the enormous burden of infectious and non-infectious diseases [19].

It has become clear that medicines are necessary, but not sufficient to offer quality services at PHCU, prevent outbreaks, and address threats such as antimicrobial resistance and the global epidemic [20]. Individual diagnosis and disease detection, prognosis and treatment planning, surveillance, and global health security are not possible without access to essential in-vitro diagnostic services [21]. As a result, without access to essential in-vitro diagnostic services at all levels of care, achieving the Sustainable Development Goals (SDGs), including universal health coverage (UHC) will not be achieved by the target year of 2030 [22].

As we have learned from global efforts to control communicable diseases such as tuberculosis and malaria, any attempts to reduce disease burden and decrease premature mortality rates will not succeed unless clinicians have access to essential in-vitro diagnostic services necessary for diagnosis, prognosis, and guidance of therapy [21]. However, while few works of literature on in-vitro laboratory services focus on addressing generalizable testing issues only in the context of a specific disease [17, 18], access to essential in-vitro diagnostics services has received little attention in SSA like Ethiopia. Thus, this study has the objective of assessing essential in-vitro laboratory services provided at PHCU in the Guragae Zone, South Ethiopia.

## Methods

### Study design and setting

Health facility-based cross-sectional study was carried out between May and July 2019. The study was conducted in the PHCU level of Gurage Zone, South Ethiopia which is one of the Zones in the Southern nation’s nationalities and people’s regional state of Ethiopia. Wolkite town is the capital of the Zone, which is located at 155 km south from Addis Ababa. Based on the 2007 Ethiopian national population and housing census [23], the total population of the Zone is projected to be about 1,609,908 with 51.38% females. There are 69 functional primary health care units in the Zone. The objectives, as well as the nature of the study, were explained to each selected PHCU after a letter of permission was obtained from Guragea Zone health department and 21 PHCU were included in this study.

### Sample size determination and procedure

The sample size was determined based on a suggested rule of thumb [24], accordingly, 21 PHCU (five primary hospitals and seventeen health care centers) were recruited from thirteen Woredas’(*administration unit within the zone in Ethiopia)* and two town administration of the Guragae Zone using probability proportional to population size to address 30% of the source population. Each PHCU was visited with a WHO checklist by a trained data collector to assess the availability of essential diagnostics service provision and laboratory equipment. The checklist has a section that included: general information about the facilities, information on essential laboratory services provided, major laboratory equipment and consumables as per WHO essential lists guideline [25]. In addition, the checklist consists of in-vitro diagnostic tests grouped by test discipline like clinical chemistry, serology, haematology, microbiology and mycology that can be used for routine patient care as well as for the detection and diagnosis of a wide array of disease conditions including communicable and NCDs.

### Data quality and analysis

Data collectors obtained training in data collection and procedure prior to data collection. The collected data were checked daily for its quality by reviewing the checklist for coherence and completeness. The collected data were entered, cleaned and analyzed using Statistical Package for Social Sciences (SPSS) version-21 (Chicago, USA). The proportion of available in-vitro diagnostics services was calculated in line with WHO in-vitro essential diagnostic lists. Results were presented as percentages using tables and figures.

## Result

### Availability and type of essential in-vitro laboratory diagnostics service

**Fig. 1:**
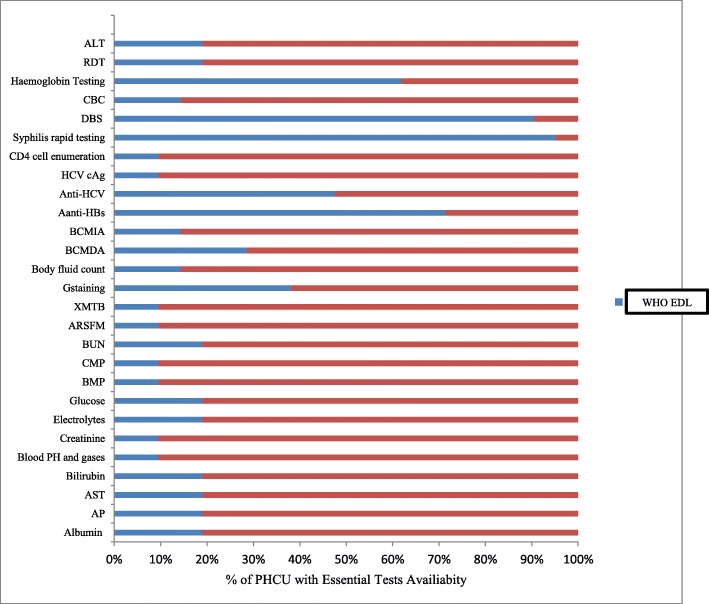
WHO Essential In Vitro Laboratory Tests in Guragae Zone Primary Health Care Unit, Southern Ethiopia, 2019. ASP-Aspatate amino-transaminase, AP-Alkaline Phosphatase, BMP-Basic metabolic panel, CMP-Comprehensive Metabolic Panel, BUN-Blood Urea Nitrogen, ARSFM-Auramine Rhodamine stain for Fluorescent Microscopy, XMTP-Xpert MTB/RIF, BCMIM-Blood Cross Matching by Indirect Agglutination, anti-HBs- Antibodis to hepatitis B surface antigen, anti-HCV**-**Antibodies to hepatitis C, HCV cAg **–**hepatitis C core antigen, DBS-Dried Blood Spot Collection, CBC-Complete Blood Count, ALT-Alanine aminotransferase, RDT-Malaria rapid diagnostic testing

Twenty-one primary care units (5 primary hospitals and 19 health care centers) in all Woreda and town administration of Guragae Zone were assessed between May and July 2019. Our results illustrated in Fig. 1 that all PHCU had major gaps in essential diagnostic test availability. Among essential in-vitro diagnostics tests listed in WHO guideline; C-reactive protein, lipid profile, Amylase and Lipase, Troponin T/I, hepatitis B e-antigen, IgM-specific antibodies to hepatitis B core antigen, Prothrombin time test and International Normalized Ratio (PT/INR), Glucose-6-phosphate dehydrogenase activity, HIV viral load, Cryptococcal antigen test, and culture test were not available in any of the surveyed PHCU with the other essential diagnostic tests availability varied a lot. However, urinalysis, urine dipstick testing, random blood sugar, malaria smear microscopy, general wet mount, Ziehl Neelsen stain, ABO-RH blood grouping testing, urine rapid test for pregnancy, hepatitis B surface antigen, antibody to HIV-1/2, and haematocrit test service were provided in all PHCU level.

### Major laboratory equipment and supply for laboratory consumables

Of the surveyed facilities, all PHCU had at least one electric binocular microscope, glucometer, centrifuge, and refrigerator. Otherwise, all PHCU had limited or no availability of the major laboratory equipment used for essential diagnostic service provision as shown in Table 1. On the other hand, of the surveyed facilities with laboratory services, twelve (57.2%) of PHCU reported stock problems on maintaining main supplies for essential laboratory services provision. In addition, the survey indicated that all health care units were with the supply of syphilis rapid test kit, urine pregnancy test kit, urine dipstick test kit, glucometer test strip, and staining solution for malaria. Six (28.6%) of the surveyed facilities were with at least one available malaria rapid diagnostic test kit while none of the facilities surveyed had the kit for cryptococcal antigen tests (Table 2).

**Table 1:** Major laboratory equipment in Guragae Zone Primary Health Care Unit, Southern Ethiopia, 2019

Equipment	Number of facilities with functional equipment	Number of facilities with non-functional equipment	Number of facilities without equipment
Light Microscope	21	–	–
Glass slides and Cover slides not reused	15/21(71.4%)	6(28.6%)	–
Refrigerator	21/21	–	–
Glucometer	21/21	–	–
Haemoglobinometer	2/21(9.5%)	9(42.9%)	10(47.6%)
Heamacue	2/21(9.5%)	–	19(90.5%)
Incubator	6/21(28.6%)	6(28.6%)	9(42.9%)
Fluorescent Microscope	2/21(9.5%)	–	19(90.5%)
Xpert machine	1/21(4.8%)	–	20(90.5%)
Chemistry analyser	5/21(23.8%)	2(9.5%)	14(66.7%)
Centrifuge	21/21	–	–
Haematology analyzer	3/21(14.3%)	–	18(85.7%)
CD4 Counter	1/21(4.8%)	–	20(90.5%)
WBC Chamber	4/21(19%)	14(66.7%)	3(14.3%)
Vortex Mixer	2/21(9.5%)	–	19(90.5%)

**Table 2:** Laboratory consumables and reagents in Guragae Zone Primary Health Care Unit, Southern Ethiopia, 2019

Laboratory consumables	Facilities with at least one available	Facilities with at least one available but expired	Not available
Malaria rapid diagnostic test Kit	6/21(28.6%)	–	15(71.4%)
Syphilis rapid test Kit	21/21	–	–
Urine Pregnancy Test Kit	21/21	–	–
Urine Dipstick test kit	21/21	–	–
DBS* test KIT	21/21	–	–
Glucometer test strip	21/21	–	–
Staining solution for malaria	21/21	–	–
HIV-1/2 antibody test Kit	20/21(95.2%)	1(4.8%)	
Cartridge	1/21(4.8%)	–	20(95.2%)
LFT test kit	3/21(14.3%)	2(9.5%)	16(76.2%)
RFT* test Kit	3/21(14.3%)	2(9.5%)	16(76.2%)
Stains Solution for CBC and differential	7/21(33.3%)	–	14(66.7%)
CD4 test kit	1/21(4.8%)	–	20(95.2%)
Kit Electrolytes	2/21(9.5%)		19(90.5%)
Gram staining Solution	8/21(38.1%)	5(23.8%)	8(38.1%)
Kit for Cryptococcal antigen test	–	–	21
Pastor Pipettes(Micro)	21	–	–
	Less than 7 days	7–14 days	Greater than 14 days
Stock out	12/21(57.2%)	5/21(23.8%)	4/21(19%)

*DBS- Dried Blood Spot, RFT-Renal Function Tests

## Discussion

While access to essential medicines is explicit in universal health coverage, access to essential diagnostics has received little attention. It has become clear that medicines are necessary, but not sufficient to offer quality primary care, prevent outbreaks, and address threats such as antimicrobial resistance and the global epidemic [20]. Diagnosis is the first critical step to offer quality healthcare and to contain emergencies. Although the WHO essential diagnostic lists (EDL) is a critical step in the right direction, the impact of the EDL will be enhanced when countries adopt the EDL to their own national needs and put in place mechanisms to implement the EDL [26].

WHO published the first EDL in May 2018 [27], it defines essential diagnostics as those that satisfy the priority health care needs of the population and are selected with due regard to disease prevalence and public health relevance. The EDL contained 113 tests; of which, 58 were considered general laboratory tests and 55 disease-specific tests with public health importance covering HIV, TB, malaria, hepatitis B and C, syphilis, and human papillomavirus [20]. WHO highlighted the fact that diagnostics are essential components for UHC, to address health emergencies, and to promote healthier populations. Moreover, the EDL offers countries a benchmark that they can use to measure and improve diagnostic services. Hence, the present study has its own importance to address is the poor state of laboratories in the study area and in the country at the PHCU level reported that most surveyed facilities had limited access to EDL.

In many SSA countries, laboratory services have suffered from inattention leading to the lack of availability of essential laboratory service [28], this has led to underutilization of laboratory testing for diagnosis [8]. Thus, syndromic approaches have replaced etiologic diagnosis [29]. On the other hand, a study from the USA shows laboratory testing influences 60–70% of critical decision-making in health with PHCU laboratories performing at least 50% of all testing [30]. However, laboratory testing may influence less than 45% of medical decision-making in SSA [5]. In support of these, all of the surveyed PHCU in the present study had major gaps with regard to EDL in the study area providing less than 20% essential diagnostic tests. In agreement with this finding, a study done in Jimma, Ethiopia reported only 23.3% of the laboratories provided clinical chemistry service [31].

Strong and efficient diagnostic laboratories are capable of providing high-quality services and important for routine diagnoses, care, treatment, and proper management and surveillance of diseases [32]. However, most resource-poor countries are faced with multiple challenges including inadequate infrastructure and inadequate laboratory equipment [8], poor supply chain management for consumables and reagents, poor system for equipment maintenance [7], lack of clear laboratory policies, insufficient laboratory management skills, weak quality management systems and accreditation of laboratories [7]. In agreement with a study conducted in the Afar Regional State of Ethiopia [2], only 52.4% of laboratories with major laboratory materials and equipment, the present study also highlights that all PHCU in the study area had a limited item of the major laboratory equipment.

Physical infrastructure needed at each level for the laboratory to provide a safe and efficient work environment in which the physical space matches the equipment needed for laboratory assays [8], the assays to be performed, the supply chain of equipment and reagents to prevent stock depletion, and the provision of routine equipment maintenance [33]. This aspect is often overlooked in SSA including Ethiopia. In agreement with this fact, more than half (57.2%) of the surveyed PHCU in this study were reported stock problems on maintaining main supplies for essential laboratory services.

Generally, the findings indicate that major gaps with regard to EDL, limited items of the major laboratory equipment, and stock problems on maintaining main supplies for essential laboratory services provision. These may lead to clinicians not to rely on laboratory test results as reported elsewhere [34–36]. It has become apparent that there is an urgent need for nations to recognize that this is a crucial gap in the health-care systems. A scarcity of appropriate health technologies, particularly in-vitro diagnostics services partly responsible for the slow progress towards achieving the SDGs, including UHC [37].

## Conclusions

The fact that access to essential diagnostic tests is the first key step in improving quality of care; such study has its own efforts to enable the implementation of EDL, and improve access to diagnostics in the country. The present study will form the basis for providing essential primary care services in the study area. The responsible body in the health care system hierarchy of the country should invest in strengthening primary care units and make sure quality-assured essential diagnostic tests are available at all facilities in the study area. While the WHO EDL is a welcome development, the list, by itself, will not have an impact, unless countries adopt and adapt the WHO list, develop their own national lists, and put in place mechanisms to improve access to tests on the EDL. The authors also believe that countries like Ethiopia should adopt the WHO EDL and make essential diagnostic tests accessible at PHCU level within the framework of UHC.

## Data Availability

The datasets used and/or analyzed during the current study are available from the corresponding author on reasonable request.

## Abbreviations

EDL: Essential Diagnostic Lists
NCDs: Non Communicable Diseases
PHCU: Primary Health Care Unit
SDGs: Sustainable Development Goals
SSA: Sub Saharan Country
UHC: Universal Health Coverage
WHO: World Health Organization
